# Metastatic behavior and overall survival according to breast cancer subtypes in stage IV inflammatory breast cancer

**DOI:** 10.1186/s13058-019-1201-5

**Published:** 2019-10-17

**Authors:** D. J. P. van Uden, M. C. van Maaren, L. J. A. Strobbe, P. Bult, J. J. van der Hoeven, S. Siesling, J. H. W. de Wilt, C. F. J. M. Blanken-Peeters

**Affiliations:** 1 Department of Surgery, Radboud University Medical Center Nijmegen, Geert Grooteplein Zuid 10, 6525 GA Nijmegen, The Netherlands; 2 Department of Research, Netherlands Comprehensive Cancer Organisation (IKNL), Hoog Catharijne, Godebaldkwartier 419, 3511 DT Utrecht, The Netherlands; 3 Department of Health Technology and Services Research, University of Twente, Drienerlolaan 5, 7522 NB Enschede, The Netherlands; 4 Department of Surgery, Canisius Wilhelmina Hospital, Weg door Jonkerbos 100, 6532 SZ Nijmegen, The Netherlands; 5 Department of Pathology, Radboud University Medical Center Nijmegen, Geert Grooteplein Zuid 10, 6525 GA Nijmegen, The Netherlands; 6 Department of Medical Oncology, Radboud University Medical Center Nijmegen, Geert Grooteplein Zuid 10, 6525 GA Nijmegen, The Netherlands; 7 Department of Surgery, Rijnstate Hospital, Wagnerlaan 55, 6815 AD Arnhem, The Netherlands

**Keywords:** Inflammatory breast cancer, Breast cancer subtype, Survival, Metastases

## Abstract

**Background:**

Distant metastatic disease is frequently observed in inflammatory breast cancer (IBC), with a poor prognosis as a consequence. The aim of this study was to analyze the association of hormone receptor (HR) and human epidermal growth factor receptor-2 (HER2) based breast cancer subtypes in stage IV inflammatory breast cancer (IBC) with preferential site of distant metastases and overall survival (OS).

**Methods:**

For patients with stage IV IBC, diagnosed in the Netherlands between 2005 and 2016, tumors were classified into four breast cancer subtypes: HR+/HER2−, HR+/HER2+, HR−/HER2+, and HR−/HER2−. Patient, tumor, and treatment characteristics and sites of metastases were compared. OS of the subtypes was compared using Kaplan-Meier curves and the log-rank test. Association between subtype and OS was assessed in multivariable models using logistic regression.

**Results:**

In total, 744 eligible patients were included: 340 (45.7%) tumors were HR+/HER2−, 148 (19.9%) HR−/HER2+, 131 (17.6%) HR+/HER2+, and 125 (16.8%) HR−/HER2−. Bone was the most common metastatic site in all subtypes. A significant predominance of bone metastases was found in HR+/HER2− IBC (71.5%), and liver and lung metastases in the HR−/HER2+ (41.2%) and HR−/HER2− (40.8%) subtypes, respectively. In multivariable analysis, the HR−/HER2− subtype was associated with significantly worse OS as compared to the other subtypes.

**Conclusion:**

Breast cancer subtypes in stage IV IBC are associated with distinct patterns of metastatic spread and display notable differences in OS. The use of breast cancer subtypes can guide a more patient-tailored staging directed to metastatic site and extend of disease.

## Introduction

Inflammatory breast cancer (IBC) has the clinical appearance of inflammation of the breast with pathological evidence of malignancy. It comprises 1% of all breast cancers and is the most aggressive form of breast cancer [1].

Breast cancer in general can be categorized into four subtypes based on immunohistochemistry of the hormone receptors (HR), subdivided in estrogen receptor (ER) and progesterone receptor (PR), and human epidermal growth factor receptor-2 (HER2) [2]. HER2-enriched (HR−/HER2+) and triple negative (HR−/HER2−) tumors in non-IBC have a worse breast cancer-specific survival in comparison with the other subtypes, although the introduction of targeted therapy for HER2-positive breast cancer has increased survival for this subtype [3, 4].

We recently demonstrated that HR/HER2-based breast cancer subtypes influence prognosis and treatment response in patients with IBC without distant metastases [5]. However, nearly 40% of patients with IBC are diagnosed with synchronous distant metastases (stage IV disease), and it is unknown what role the HR/HER2-based subtypes play in this stage [6].

Besides histological subtype, site of metastases at time of diagnosis also strongly influences prognosis of metastatic breast cancer, with bone metastases having a better prognosis compared to lung and liver metastases [7]. In patients with stage IV IBC as initial presentation, the correlation between breast cancer subtypes on both the preferential site of metastases and on OS has not been evaluated before.

While progress has been made in recent years, the survival of IBC remains poor. Both the rarity and the aggressiveness contribute to the difficulty in treating IBC [1]. Improving the understanding of distinct patterns of metastatic spread hopefully will lead to a better understanding of this fatal disease. Moreover, it might influence the diagnostic process for patients presenting with IBC and may be supportive to the multidisciplinary discussion which therapies are appropriate once distant disease has been diagnosed. The purpose of this study was to determine the association of breast cancer subtypes (HR/HER2-based) on preferential site of metastatic disease and overall survival (OS) in patients presenting with stage IV IBC.

## Materials and methods

### Data source

The most important data on cancer in the Netherlands are registered in the nationwide population-based Netherlands Cancer Registry (NCR), hosted by the Netherlands Comprehensive Cancer Organisation (IKNL). The NCR registers all newly diagnosed malignancies in the Netherlands, using the nationwide network and registry of histo- and cytopathology in the Netherlands (PALGA) as main source of notification. Trained registrars from the IKNL directly collect data from the patient’s medical records. Morphology and differentiation are coded according to the International Classification of Diseases for Oncology (ICD-O), third edition [8]. Staging is coded according to the Tumor, Node and Metastasis (TNM) classification system. The specific edition depended on the year of incidence [9, 10]. With respect to IBC, the criteria used in the TNM system have not changed over time. Yearly linkage to the municipal administration database is used to verify the patient’s vital status and, if applicable, date of death. Follow-up has been completed until December 31, 2016. The privacy committee of the NCR has approved this study.

### Patients and study variables

Patients, diagnosed from 2005 to 2016, with clinical T4dN0–3 M0 breast cancer were identified: diffuse erythema and edema (peau d’orange) involving a third or more of the skin of the breast. Patients with only a pathological T4d status without clinical T4d status were excluded. Patients were classified into four breast cancer subtypes, based on HR/HER2-status: HR+ (ER+ and/or PR+)/HER2−, HR+(ER+ and/or PR+)/HER2+, HR− (ER− and PR−)/HER2+, and HR− (ER− and PR−)/HER2−. Patients were excluded when data on HR and/or HER2 status were missing.

According to Dutch guidelines, ER/PR status had been determined with immunohistochemistry (IHC). At least 10% positive tumor nuclei were considered as a positive result. In the Netherlands, HER2 status was considered positive with an immunohistochemical score of 3+ (at least 10% of tumor cells with strong complete membrane staining) or amplification of the HER2 gene diagnosed with in situ hybridization (ISH) (at least 10% of tumor cells showing a ratio of HER2 probe to centromere chromosome 17 probe of > 2.2 or with single probe HER2 test when mean > 6 HER2 genes per tumor nucleus were detected) or with other amplification-based techniques, such as multiplex ligation-dependent probe amplification (MLPA). In case of an immunohistochemical score of 2+ (at least 10% of tumor cells with slight to moderate complete membrane staining; considered as an equivocal result), ISH or MLPA was performed. If in this case HER2 was found to be amplified, HER2 was considered positive. HER2 status was considered negative with an immunohistochemical score of 0 or 1+ or if ISH or MLPA showed no amplification of the HER2 gene. In the Netherlands, some variation in determining the HER2 status existed in the period 2005–2016 (especially the cutoff for amplification (> 2.2 or ≥ 2) in the double probe ISH test). For this study, the HER2 status as was registered in the NCR was used.

Metastases diagnosed within 3 months after the date of determination of the treatment plan were considered to be synchronous with the primary tumor and incorporated in initial staging. Different sites of metastases were analyzed: bone, lung, liver, and other and multiple organs affected.

Treatment modalities were analyzed. The use of trimodality treatment (combination of subsequent neoadjuvant chemotherapy, surgery, and adjuvant locoregional radiation therapy) was evaluated. Chemotherapy, endocrine therapy, and targeted therapy (trastuzumab) were reported as administered or not administered.

### Statistical analysis

Tumor characteristics, site and number of metastasis, and treatment were compared between the different HR and HER2 subgroups using chi-squared tests for categorical variables and non-parametric approaches (Mann-Whitney *U* tests) for continuous variables. Fisher’s exact test was used to determine if non-random associations between two categorical variables in case of less than five patients per stratum existed. The *p* value was not calculated in case there were 0 cases in one or more strata. Follow-up was calculated until time of death or end of observation. OS was determined using Kaplan-Meier curves and breast cancer subtypes, and tumor localizations were compared using the log-rank test. To adjust for patient, tumor, and treatment-related characteristics, a multivariable Cox proportional hazard analysis was performed. Variables included were age, breast cancer subtype, nodal stage, histological tumor type and grade, and trimodality therapy, as these variables were significantly different between breast cancer subtypes and significantly influenced the outcome (*p* < 0.1). For all other analyses, a *p* value < 0.05 was considered as statistically significant.

## Results

A total of 2235 patients with IBC were diagnosed in the Netherlands between January 2005 and December 2016 of whom 842 patients presented with stage IV IBC (33.3%) at diagnosis. Of these 842 patients, 98 patients were excluded due to an unknown HR or HER2 status, leaving 744 patients for inclusion in the present study. The 98 excluded patients with an unknown HR/HER2 less often underwent any form of treatment and were significantly older (data not shown). In 2005, the first year of registration in the database, 17.3% of patients had an unknown receptor status, but this was in later years low (range 4.1–11.2%). From 2005 onwards, the incidence of stage IV IBC is increasing (data not shown).

### Breast cancer subtypes and tumor characteristics

Among the eligible patients, the distribution of breast cancer subtypes was as follows: 340 (45.7%) HR+/HER2−, 148 (19.9%) HR−/HER2+, 131 (17.6%) HR+/HER2+, and 125 (16.8%) HR−/HER2−. In the HR−/HER2− subtype, grade 3 tumors were found most frequently (29.6% versus 12.9–24.3%). HR+/HER2+ and HR−/HER2+ tumors were more often found in ductal cancer (90.8 and 89.9%, respectively) and HR+/HER2− tumors in lobular cancer (14.4%) (Table 1).

**Table 1:** Patient, treatment, and tumor-related characteristics of all stage IV IBC patients per breast cancer subtype (*n* = 744)

	HR+/HER2− (*n* = 340)	HR+/HER2+ (*n* = 131)	HR−/HER2+ (*n* = 148)	HR−/HER2− (*n* = 125)	*p* value
	*N* (%)	*N* (%)	*N* (%)	*N* (%)	
Age, median (IQR)	61 (52–73)	60 (50–74)	57.5 (50–69)	62 (52–73)	0.191
Histological grade					
1	6 (1.8)	2 (1.5)	0 (0.0)	1 (0.8)	NC
2	40 (11.8)	13 (9.9)	16 (10.8)	8 (6.4)	
3	44 (12.9)	19 (14.5)	36 (24.3)	37 (29.6)	
Unknown*	250 (73.5)	97 (74.1)	96 (64.9)	79 (63.2)	
Histological type					
Ductal	279 (82.1)	119 (90.8)	113 (89.9)	110 (80.0)	*0.019*
Lobular	49 (14.4)	7 (5.3)	10 (6.7)	8 (6.4)	
Other	12 (3.5)	5 (3.8)	5 (3.4)	7 (5.6)	
Metastatic sites					
1	167 (49.1)	54 (41.2)	74 (50.0)	58 (46.4)	
2 or more	173 (50.9)	77 (58.8)	74 (50.0)	67 (53.6)	0.414
Surgery					
Yes	60 (17.7)	24 (18.3)	40 (27.0)	25 (20.0)	0.113
No	280 (82.4)	107 (81.7)	108 (73.0)	100 (80.0)	
ALND					
Yes	46 (13.5)	14 (10.7)	24 (16.2)	18 (14.4)	0.603
No	294 (86.5)	117 (89.3)	124 (83.8)	107 (85.6)	
Chemotherapy					
Yes	166 (48.8)	88 (67.2)	128 (86.5)	103 (82.4)	*< 0.001*
No	174 (51.2)	43 (32.8)	20 (13.5)	22 (17.6)	
Endocrine therapy					
Yes	253 (74.4)	83 (63.4)	6 (4.1)	2 (1.6)	*< 0.001*
No	87 (25.6)	48 (36.6)	142 (96.0)	123 (98.4)	
Radiation therapy					
Yes	65 (19.1)	21 (16.0)	28 (18.9)	31 (24.8)	0.347
No	275 (80.9)	110 (84.0)	120 (81.1)	94 (75.2)	
Anti-HER2 therapy					
Yes	21 (6.2)	90 (68.7)	111 (75.0)	11 (8.8)	*< 0.001*
No	319 (93.8)	41 (31.3)	37 (25.0)	114 (91.2)	
Trimodality therapy					
Yes	30 (8.8)	7 (5.3)	14 (9.5)	9 (7.2)	0.555
No	310 (91.2)	124 (94.7)	134 (90.5)	116 (92.8)	

*p* values indicated in italics are considered as statistically significant (*p* < 0.05). *Abbreviations*: *IQR* interquartile range, *ALND* axillary lymph node dissection, *HR* hormone receptor, *HER2* human epidermal growth factor receptor-2, *NC* not calculable.*Histological grade is usually determined postoperatively, and since most patients are not treated with surgery, this variable is unknown in most of the patients. Trimodality therapy: neoadjuvant chemotherapy, surgery, radiation therapy

### Site of metastases

In 391 patients (52.6%), metastases were found in multiple organs. In all breast cancer subtypes, bone metastases were most commonly diagnosed, with the highest percentage found in the HR+/HER2− subtype (71.5%). Lung metastases occurred significantly more often in HR−/HER2− IBC (40.8%). Liver metastases were significantly more often found in HER2-enriched (HR−/HER2+) tumors (41.2%) (Table 2). No differences were found with regard to brain metastases.

**Table 2:** Frequencies of metastatic sites, divided by molecular subtype (*n* = 744)

	HR+/HER2− (*n* = 340)	HR+/HER2+ (*n* = 131)	HR−/HER2+ (*n* = 148)	HR−/HER2− (*n* = 125)	*p* value
Type of metastasis per subtype					
Multiple sites	173 (50.9)	77 (58.8)	74 (50.0)	67 (53.6)	0.414
Only one site	167 (49.1)	54 (41.2)	74 (50.0)	58 (46.4)	
Bone	102 (30.0)	30 (22.9)	24 (16.2)	21 (16.8)	*< 0.001*
Lung	17 (5.0)	2 (1.5)	8 (5.4)	12 (9.6)	
Liver	12 (3.5)	8 (6.1)	20 (13.5)	8 (6.4)	
Other^#^	36 (10.6)	14 (10.7)	22 (14.9)	17 (13.6)	
All found metastases^$^					
Bones	243 (71.5)	90 (68.7)	75 (50.7)	52 (41.6)	*< 0.001*
Lung	102 (30.0)	41 (31.3)	35 (23.7)	51 (40.8)	*0.023*
Liver	75 (22.1)	43 (32.8)	61 (41.2)	39 (31.2)	*< 0.001*
Brain	7 (2.1)	5 (3.8)	3 (2.0)	3 (2.4)	0.713
Other/unknown	35 (10.3)	13 (9.9)	21 (14.2)	17 (13.6)	0.496

*p* values indicated in italics are considered as statistically significant (*p* < 0.05). *Abbreviations*: *HR* hormone receptor, *HER2* human epidermal growth factor receptor-2. ^#^Including brain metastasis. ^$^Cumulative percentage per subtype exceeds 100% due to the occurrence of multiple metastases at diagnosis

### Treatment

Of the 744 patients with stage IV IBC, 149 patients (20.0%) underwent breast surgery as part of their treatment. Chemotherapy was administered in 485 patients (65.2%), significantly less often in HR+/HER2− tumors compared to the other subtypes. In 253 patients (74.4%) of HR+/HER2− tumors, endocrine treatment was given. HR−/HER2+ and HR−/HER2− received chemotherapy more often compared to the other subtypes (86.5% and 82.4%, respectively). No differences were found between subtypes regarding the frequency of application of trimodality therapy. Overall, just over 70% of HER2-enriched tumors were treated with targeted therapy (Table 1).

### Survival outcomes

Median follow-up was 16.1 months (interquartile range 7.08–30.48 months), with a median OS of the entire cohort of 22.8 months (95% CI 1.68–2.03 months). No significant differences were found with regard to survival between age groups < 60 years and ≥ 60 years.

**Fig. 1:**
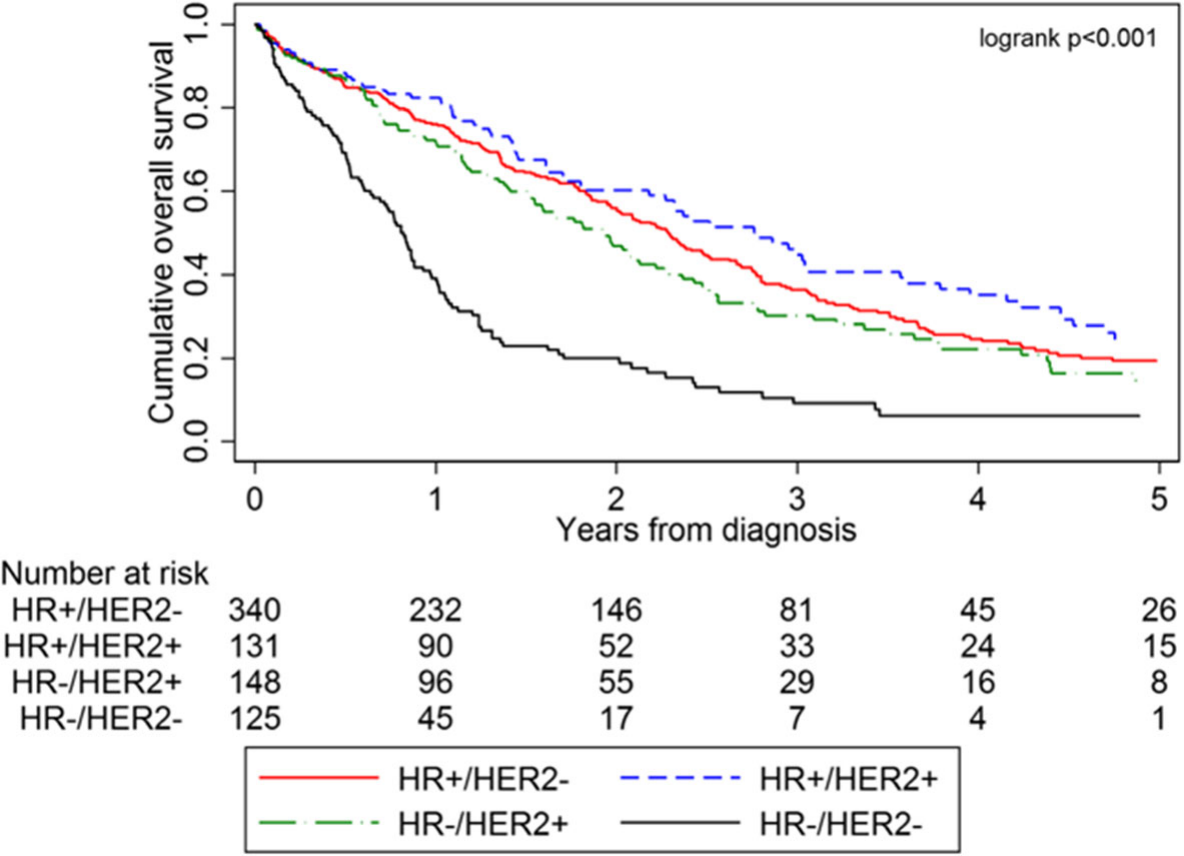
Kaplan-Meier curves displaying OS of all stage IV IBC from 2005 to 2016, presenting with stage IV at diagnosis, divided by breast cancer subtype (*n* = 744). Abbreviations: HR, hormone receptor; HER2, human epidermal growth factor receptor-2

Stage IV IBC patients with HR+/HER2+ tumors exhibited the most prolonged OS, whereas patients with HR−/HER2− tumors exhibited the worst OS (*p* < 0.001, Fig. 1): HR+/HER2− 36.5%, HR+/HER2+ 45.8%, HR−/HER2+ 31.8%, and HR−/HER2− 15.2%. Five-year OS for the entire cohort of patients was 33.6%.

**Fig. 2:**
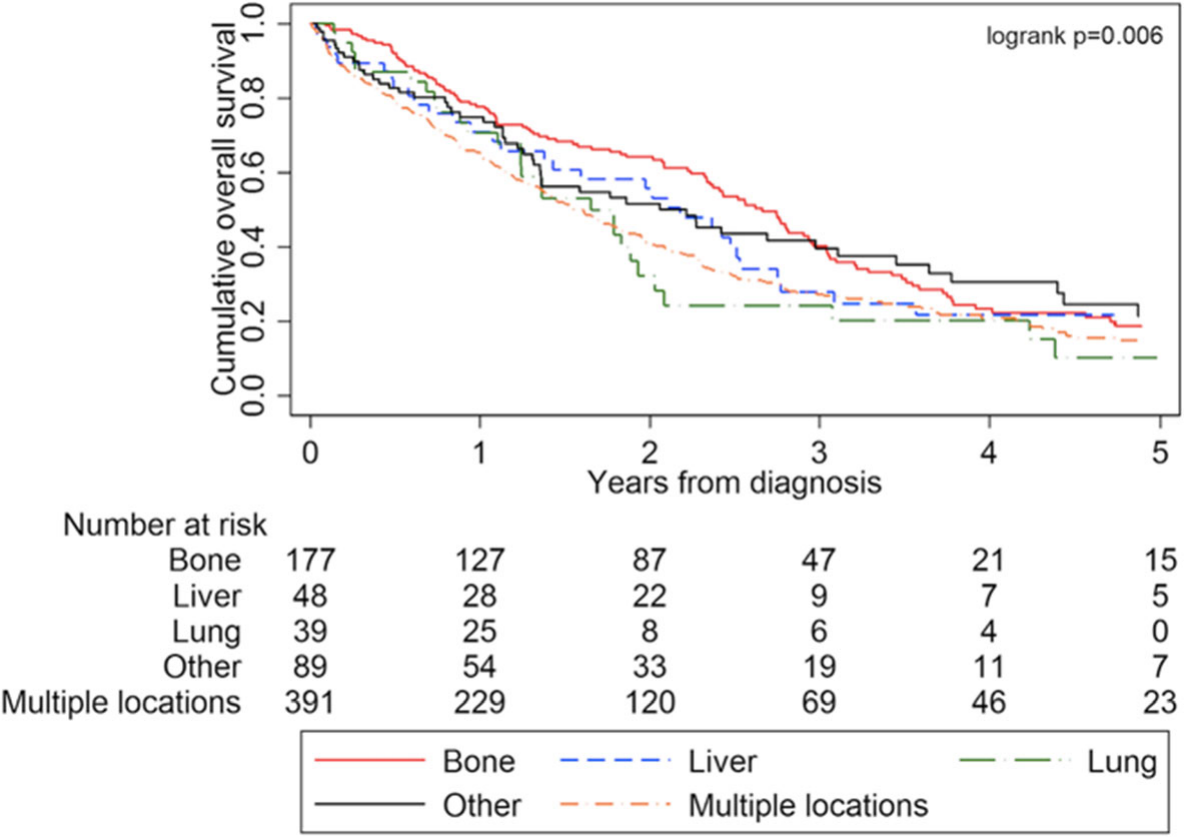
Kaplan-Meier curves displaying OS of all stage IV IBC from 2005 to 2016, presenting with stage IV at diagnosis (*n* = 744), divided by metastatic site

A worse survival was seen in case of multiple organ involvement, displayed in the Kaplan-Meier curves for OS of stage IV IBC patients according to site of distant metastasis (Fig. 2).

Multivariable analysis revealed that surgery, chemotherapy, targeted therapy, and antihormonal therapy were all independently associated with better survival (Table 3). Both HR−/HER2+ and HR−/HER2− subtypes were associated with a significantly worse OS compared to the HR+/HER2+ and HR+/HER2− subtypes. Multiple site metastases were associated with a significant worse survival (HR 1.32 [95% CI 1.04–1.68]).

**Table 3:** Adjusted hazard ratios for 5-year OS in patients with IBC presenting with stage IV disease

	Hazard ratio	(95% CI)	*p* value
Age	1.01	1.00–1.01	0.168
Year of diagnosis	0.98	0.95–1.02	0.352
Clinical nodal stage			
N0	1 (ref)		
N1	1.15	0.78–1.68	0.468
N2	1.23	0.74–2.07	0.414
N3	1.32	0.88–1.99	0.174
Surgery			
No	1 (ref)		
Yes	0.56	0.42–0.74	*< 0.001*
Chemotherapy			
Yes	1 (ref)		
No	1.62	1.24–2.14	*< 0.001*
Targeted therapy			
Yes	1 (ref)		
No	2.76	1.70–3.05	*< 0.001*
Antihormonal therapy			
Yes	1 (ref)		
No	2.16	1.62–2.89	*< 0.001*
Radiation therapy			
Yes	1 (ref)		
No	1.11	0.84–1.46	0.457
Molecular subtype			
HR+/HER2−	1 (Ref)		
HR+/HER2+	1.17	0.86–1.61	0.319
HR−/HER2+	1.59	1.12–2.24	*0.009*
HR−/HER2−	1.94	1.41–2.67	*< 0.001*
Location of metastases			
Bone only	1 (ref)		
Liver only	0.86	0.56–1.33	0.507
Lung only	1.27	0.81–1.99	0.292
Other	0.99	0.69–1.40	0.934
Multiple organs	1.32	1.04–1.68	*0.021*

*Abbreviations*: *ref*. refererence, *OR* odds ratio, *CI* confidence interval, *HR* hormone receptor, *HER2* human epidermal growth factor receptor-2. *p* values indicated in italics are considered as statistically significant (*p* < 0.05)

## Discussion

There is limited knowledge on the influence of breast cancer subtypes, based on hormone receptor status and HER2-status, on clinical outcome in stage IV IBC. This large study demonstrates that breast cancer subtypes in stage IV IBC are associated with unique patterns of distant metastatic spread and differences in OS.

To our knowledge, this is the first extensive analysis of the influence of HR/HER2-based breast cancer subtypes on the preferential site of metastases and OS in stage IV IBC. Data of previous studies were derived from patients treated in single institutions [11, 12], whereas our study contains data based on a nationwide population-based cancer registry including unselected and unbiased data of all hospitals (both academic and non-academic) in the Netherlands. Therefore, our study, which includes the largest patient number so far, represents valuable data on current clinical presentation and practice.

### Inflammatory breast cancer subtypes and preferential site of metastasis

At first presentation, patients with IBC display significantly higher rates of distant metastases compared to non-inflammatory locally advanced breast cancer (39.7% versus 34.1%) [6].

In our current cohort, 744 patients with stage IV IBC at diagnosis were evaluated, which accounted for 33.3% of all patients diagnosed with IBC in the studied period. Over 50% of patients presented with multiple metastatic sites with different frequencies of the site of distant metastases among the various breast cancer subtypes. Multivariable analysis revealed that metastatic involvement of multiple sites was independently associated with a worse survival.

Bone metastases were most commonly diagnosed in all stage IV IBC subtypes with a significant predominance in the HR+/HER2− group. Liver metastases were more frequently observed in the HER2-enriched group and lung metastases in the HR−/HER2− group. This is not in agreement with a previous SEER analysis which did not show a significant association between IBC subtypes and site of metastases, which may well be attributed to the small sample with only 83 patients with stage IV IBC analyzed in that study [13]. We used data from the NCR to demonstrate that the metastatic patterns of stage IV IBC seem rather comparable to stage IV breast cancer in general [14]. A SEER analysis demonstrated that HR+/HER2+ and HER2-enriched subtypes are prone to abdominal/pelvic metastases and HR+/HER2− and HR+/HER2+ subtypes to bone metastases, while the HR−/HER2− subtype was prone to lung/mediastinal metastases [14]. In the present study, the occurrence of brain metastases was evidently lower than previously reported by Warren et al., who advised to incorporate surveillance brain MRI after diagnosis of extracranial metastatic disease in IBC [15]. However, in their study, no statistically significant association was found between primary tumor subtype and increased risk of development of brain metastases. A US-based single-center analysis in 203 IBC patients showed that the median time to development of brain metastases was 19 months [16]. One of the reasons of the low incidence of brain metastases in our study might be the fact that in the NCR only synchronous metastases are recorded, and as such, we were not able to analyze subsequent brain metastases that occurred more than 3 months after diagnosis.

### Treatment of stage IV inflammatory breast cancer

Management of synchronous stage IV IBC includes primary systemic cytotoxic therapies and targeted HER2 therapy in case of HER2 positivity [17]. One area of debate is whether patients with stage IV IBC also should undergo local resection of the breast tumor. In the absence of prospective data, a potential survival benefit from removal of the breast tumor is suggested by retrospective evidence [11, 12, 18].

To our knowledge, there are three prospective trials conducted evaluating the effect of removal of the primary tumor in stage IV breast cancer, in which conflicting results were presented: two studies could not demonstrate a survival benefit [19, 20] and one showed an improved survival after 40 months follow-up (initially not showing any survival benefit of surgery after 36 months of follow-up) [21].

Just over 20% of all patients in our analysis underwent surgical resection of the primary tumor and just over 8% received trimodality treatment. This combination of neoadjuvant chemotherapy, surgery, and adjuvant radiation therapy is considered to be the most effective treatment regimen in stage III IBC [17]. Our numbers indicate that, also in the metastatic setting, surgery is used for locoregional management.

### Inflammatory breast cancer subtypes and survival

An important finding of the present study is the highly variable prognosis among the different breast cancer subtypes in stage IV IBC. Patients with HR+/HER2+ IBC had the best survival among the four subgroups, whereas HR−/HER2− IBC is an independent prognostic factor for decreased survival, compared to the other subtypes. These results are consistent with previous studies in stage III IBC as well as stage IV non-IBC, both showing that HR−/HER2− tumors have the worst prognosis [5, 22, 23]. The 5-year OS of HR+/HER2+ IBC patients is 3.5-fold higher than that of patients with HR−/HER2− IBC, whereas HR+/HER2− and HR−/HER2+ subtypes display similar survival rates to each other but evidently lower compared to the HR+/HER2+ subtype. These ratios are comparable to a recent SEER analysis of metastatic breast cancer. Improved survival of the HR+/HER2+ subtype most likely reflects the use of HER2-targeted therapies. After the development of HER2-targeted therapies for the treatment of metastatic breast cancer in general, the survival of patients with HER2-positive tumors was greatly improved. This effect was irrespective of the HR status of the tumor [24]. Data were collected from patients who presented with stage IV IBC in the period 2005–2016. The last years, trastuzumab emtansine and pertuzumab enlarged the therapeutic arsenal for HER-2 positive patients, and as a consequence, the prognosis for survival will be even better nowadays than in the study period [25].

In general, the present study confirmed that compared with other primary tumor characteristics, breast cancer subtypes based on the HR and HER2 status of the primary tumor in stage IV IBC are important predictors of OS.

Several limitations of the present study have to be discussed. Firstly, the NCR does not register cause of death, and therefore, breast cancer-specific survival could not be determined. However, since all included patients already were diagnosed with stage IV disease at diagnosis, and since metastatic disease is the major cause of cancer-related deaths among breast cancer patients, the cause of death in our population is most likely to be breast cancer specific [26]. Secondly, it should be noted that 98 patients who were excluded with an unknown HR/HER2 less often underwent any form of treatment. These patients were significantly older and represent a specific subgroup of stage IV IBC patients. Reasons why older patients with cancer accept or decline treatment vary considerably, but the most consistent determinant found in the literature is physician recommendation [27]. Unfortunately, we are not able to draw firm conclusions on the absence of HR/HER2 data, since reasons for the waiver of treatment modalities could not be investigated in this database. These factors, as well as comorbidity, were not registered in the NCR and could not be accounted for in our study. Moreover, local therapy of the sites of metastatic disease (for example, resection of metastases and/or radiation therapy) could not be analyzed in this study.

We chose to only analyze clinical T4d breast cancers, instead of analyzing both clinical and pathological T4d breast cancers. However, since IBC is typically diagnosed clinically (dermal lymphatic invasion without typical clinical findings is not sufficient for a diagnosis of IBC), analysis of clinical T4d breast cancers seems to be the most accurate approach. As with any information obtained retrospectively from the abstraction of medical records, we acknowledge the dependency on the availability of data and reporting accuracy.

Furthermore, a central pathology review during treatment was not conducted, which might have led to an altered HR/HER2 status in several patients. Therefore, the potential impact of inter-institutional discordance was not evaluated. However, our current analysis reflects daily clinical practice in which local laboratories do not send all samples to a central laboratory and limited discordance was found in previous analyses which addressed the possibility of potential discordance [28–30]. Furthermore, discordances in ER/PR/HER2 test results between tumor core needle biopsy taken at the time of diagnosis and tumor resection material are low, also in patients receiving any form of neoadjuvant therapy [31].

Finally, information regarding the type of diagnostic modalities is lacking. Given the high rate of metastatic disease at presentation, patients with IBC undergo extensive staging, including whole body bone scintigraphy, ultrasonography of the liver, and a chest X-ray. Some institutions might have used other modalities such as 18-fluorodeoxyglucose positron emission tomography/computed tomography (PET/CT), which might influence the detection of distant metastases compared to traditional modalities [32]. More than 20% of patients appear to have distant metastases after FDG-PET scanning in comparison with conventional staging in locally advanced breast cancer [33]. However, with regard to subtypes, no difference is expected, since there is no guideline of which breast cancer subtype should receive a specific type of diagnostic modality and a potential diagnostic bias would be present for all breast cancer subtypes and this will not affect the differences we report.

### Clinical relevance

IBC is diagnosed at a younger age with survival rates which are clearly inferior to the average rates for patients with non-IBC [6]. Similar to IBC in general, the incidence of stage IV IBC seems to be increasing (data not shown) [6]. This might, among others, be due to increased use of improved staging modalities [32]. Knowledge of the biology of IBC has to increase to achieve improvement on the treatment of IBC. This applies for both stage III and IV IBC. Stratification of breast cancer subtypes in stage IV IBC is of clinical use for estimating prognosis, since OS differed significantly between the subtypes with the worst OS for HR−/HER2− IBC. These data might aid physicians in patient counseling regarding prognosis and underscribe the need of new systemic (targeted) therapies to improve OS in stage IV IBC and HR−/HER2− disease in particular.

Moreover, the differences seen in sites of metastases between breast cancer subtypes can guide a more patient-tailored staging directed to metastatic sites. Since metastatic disease remains the principal cause of cancer-related deaths [34], this tailored staging could lead to the identification of more effective prognostication and, hopefully in the future, individualized targeted approaches to treat these patients. Some evidence suggests a potential role for metastasis-specific local treatment (e.g., metastasectomy and radiation therapy) in the prolongation of survival, especially in oligometastatic disease, although prospective data are lacking [35]. Consequently, in patients with multiple organ metastases, locoregional treatment of metastases should be discussed and potentially omitted. This will prevent potential morbidity of non-beneficial treatments [36].

## Conclusion

This study demonstrates important differences in distant metastatic behavior and overall survival between breast cancer subtypes, as defined by HR/HER2 status, and contributes to an expanding knowledge of prognostic markers in stage IV IBC. Consequently, more focused and patient-tailored staging should be based on breast cancer subtypes in order to achieve the most accurate information on the site and extend of disease and to discuss potential treatment options in case of metastatic disease.

## Data Availability

The data that support the findings of this study are available from the NCR but restrictions apply to the availability of these data, which were used under license for the current study, and so are not publicly available. Data are however available from the authors upon reasonable request and with permission of the NCR.
